# Evaluation of Myelin Radiotracers in the Lysolecithin Rat Model of Focal Demyelination: Beware of Pitfalls!

**DOI:** 10.1155/2019/9294586

**Published:** 2019-05-29

**Authors:** Min Zhang, Gaëlle Hugon, Caroline Bouillot, Radu Bolbos, Jean-Baptiste Langlois, Thierry Billard, Frédéric Bonnefoi, Biao Li, Luc Zimmer, Fabien Chauveau

**Affiliations:** ^1^University of Lyon, Lyon Neuroscience Research Center (CRNL), Lyon, France; ^2^CNRS UMR5292, INSERM U1028, University of Lyon 1, F-69003 Lyon, France; ^3^Shanghai Jiao Tong University, School of Medicine, Department of Nuclear Medicine, Rui Jin Hospital, Shanghai, China; ^4^CERMEP-Imagerie Du Vivant, F-69677 Bron, France; ^5^University of Lyon, Institute of Chemistry and Biochemistry (ICBMS), Lyon, France; ^6^CNRS UMR5246, University of Lyon 1, F-69622 Lyon, France; ^7^Hospices Civils de Lyon, F-69677 Bron, France

## Abstract

The observation that amyloid radiotracers developed for Alzheimer's disease bind to cerebral white matter paved the road to nuclear imaging of myelin in multiple sclerosis. The lysolecithin (lysophosphatidylcholine (LPC)) rat model of demyelination proved useful in evaluating and comparing candidate radiotracers to target myelin. Focal demyelination following stereotaxic LPC injection is larger than lesions observed in experimental autoimmune encephalitis models and is followed by spontaneous progressive remyelination. Moreover, the contralateral hemisphere may serve as an internal control in a given animal. However, demyelination can be accompanied by concurrent focal necrosis and/or adjacent ventricle dilation. The influence of these side effects on imaging findings has never been carefully assessed. The present study describes an optimization of the LPC model and highlights the use of MRI for controlling the variability and pitfalls of the model. The prototypical amyloid radiotracer [^11^C]PIB was used to show that *in vivo* PET does not provide sufficient sensitivity to reliably track myelin changes and may be sensitive to LPC side effects instead of demyelination as such. *Ex vivo* autoradiography with a fluorine radiotracer should be preferred, to adequately evaluate and compare radiotracers for the assessment of myelin content.

## 1. Introduction

Multiple sclerosis (MS) is a chronic inflammatory demyelinating disorder affecting the quality of life, employment, and social relationships of approximately 2.1 million people worldwide. The formation of focal demyelinated lesions and progressive failure of remyelination is the main characteristic of MS and further leads to axonal injury and neuron loss [1]. Magnetic resonance imaging (MRI) is essential for diagnosis and continuous management of MS [2]. However, conventional MRI measurements (lesion burden, location, and type) correlate poorly with disability and lack long-term prognostic value. New disease-modifying treatments which promote remyelination are now entering clinical evaluation [3]. Therefore, an urgent challenge is to identify the best objective, reliable, and predictive biomarker of remyelination. There is no consensus on which imaging technique should be used. Advanced MRI techniques such as magnetization transfer imaging (MTI) [4] or myelin water fraction (MWF) [5] are increasingly popular as research tools but have not yet been standardized for widespread clinical application. Quantification is not straightforward, as myelin content is inferred indirectly from water binding to lipid bilayer macromolecules [6, 7].

By contrast, positron emission tomography (PET) may provide more direct quantitative assessment of myelin content, by injection of a radiolabeled probe targeting myelin proteins. Several independent groups have illustrated the ability of [^11^C]PIB to detect white matter alterations in MS [8, 9] and other pathological conditions [10, 11]. These pioneering studies have stimulated the search for new myelin radiotracers with enhanced specific (white matter) binding ratio over nonspecific (gray matter) binding ratio [12] and, ideally, with fluorine-18 labeling to enable wider clinical use [13, 14]. Among available models of demyelination, the simple model consisting in intracerebral injection of lysophosphatidylcholine (LPC, or lysolecithin) [15] appears attractive for first-step evaluation of radiotracers: the detergent action of LPC produces focal demyelination, followed by spontaneous progressive remyelination, while the contralateral hemisphere may serve as an internal control in a given animal. Hence, several imaging studies have used the LPC model in rats, to evaluate MRI biomarkers [16, 17] or PET radiotracers [12, 18]. However, as previously reported by several groups, LPC-induced demyelination may be associated with concurrent focal necrosis [16, 18, 19] and/or ventricle dilation [20]. The influence of these side effects on imaging findings has never been carefully assessed.

The present study describes an optimization of the LPC model and highlights the use of MRI for controlling the variability and pitfalls of the model. Using the prototypical radiotracers [^11^C]PIB and [^18^F]AV-45, we show that (i) *in vivo* PET does not provide sufficient sensitivity to reliably track myelin changes in this model and (ii) *ex vivo* autoradiography should rather be used to adequately evaluate and compare radiotracer performance.

## 2. Materials and Methods

### 2.1. Animals

A completed ARRIVE (Animal Research: Reporting of In Vivo Experiments) guidelines checklist is included as supplementary material (S1). All experiments were carried out under a protocol approved by the local ethical review board (“Comité d'éthique pour l'Expérimentation Animale Neurosciences Lyon”, registration code: C2EA-42), authorized by the French Ministry of Higher Education and Research (n°5892-2016063014207327v2), and were in accordance with European directives on the protection and use of laboratory animals (Council Directive 2010/63/UE, French decree 2013-118).

### 2.2. Housing

Adult male Sprague Dawley rats (ILAR code Crl:CD(SD)) were ordered from Charles River (L'Arbresle, France) and given a minimum of five days to acclimate to the conventional housing facility, under temperature-controlled (range 20–24°C) conditions and a 12 : 12 h light-dark cycle, with lights on at 07 : 00 and off at 19 : 00. Animals were housed by group of six in open polycarbonate cages (Tecniplast, 2000P, *L* × *W* × *H* = 610 × 435 × 215 mm, floor area 2065 cm^2^), with stainless steel lids. Environmental enrichment included spruce-based bedding of 2–4 mm granulometry (Lignocel 3/4 s), round tinted polycarbonate tunnels (153 × 75 mm, SERLAB), and hazel chew blocks (JR Farm). Animals were given access to pellets of wheat and corn (Teklad Global 18% Protein Rodent Diet, ENVIGO) and tap water ad libitum. During housing, animals were monitored daily for health status. At the start of the experiments, animals weighed 250–350 grams.

### 2.3. Surgery

Demyelination was induced by stereotaxic injection of LPC (Sigma-Aldrich, ref. L4129) at 1% in saline solution into the right corpus callosum and saline into the contralateral site, infused at 0.1 *μ*l/min. Three different injection conditions were successively tested (no randomization):

- In group 1 (*n*=8), injection sites were adapted from previous studies: AP −0.3 mm; ML ±3.0 mm; DV −3.5/−4.0/−4.5/−5.0 mm; 2.5 *μ*l each, from depth to superﬁcial
- In group 2 (*n*=9), injection sites were slightly adjusted: AP −0.3 mm; ML ±3.3 mm; DV −3.0/−3.7/−4.3/−5.0 mm; 2.5 *μ*l each, from depth to superﬁcial
- In group 3 (*n*=10), injection sites were restricted to corpus callosum: AP −0.3 mm; ML ±3.3 mm; DV −2.8/−3.5 mm; 2.5 *μ*l each, from depth to superﬁcial

Rats were anesthetized with isoﬂurane inhalation in air in an anesthesia induction box and then transferred to a stereotaxic apparatus (Stoelting) equipped with a mask delivering isoﬂurane at 1.0–2.5% for the duration of the experiment. Body temperature was maintained by a heating pad set at 37°C and monitored using a rectal probe. Pain was controlled by buprenorphine (Buprecare, Axience), a potent opioid analgesic, injected subcutaneously at a dose of 0.05 mg/kg 20 min before any surgical act was performed. A local analgesic (lidocaine/prilocaine 5%, Pierre Fabre) was also applied on the scalp before incision. After bilateral craniotomy, LPC and saline were slowly infused with 30-gauge needles (RN type, NH-BIO) via a tubing (Fine Bore Polythene Tubing, Portex, Smith Medical Intl) connected to syringes installed in injection pumps (World Precision Instruments). The needles were left in place for 2 min and then slowly withdrawn. After injection, the scalp was sutured, and an antiseptic (povidone-iodine) and local analgesic (lidocaine) were applied. The rats were then allowed to recover from anesthesia. The long-term action of buprenorphine (ca 6 hours) allowed the animals to completely recover without the need of a second administration. No adverse events were observed.

Imaging studies were performed on an additional batch of animals (10 animals injected as in group 3, with a single animal being the experimental unit) and started 7 days postinjection with MRI. One animal showed no MRI changes and was excluded. *In vivo* PET, or *ex vivo* autoradiography, was performed between 8 and 15 days postinjection in 9 animals, a period during which no significant spontaneous remyelination is expected [12, 17, 18].

### 2.4. *In vivo* Study

All imaging sessions were performed under isoflurane anesthesia delivered in air by approved systems (TEM Sega).

#### 2.4.1. MR Imaging

The animals were placed in prone position in a dedicated plastic bed equipped with a stereotactic holder (Bruker Biospec Animal Handling Systems) and maintained under gaseous anesthesia delivered via a cone mask throughout the MRI protocol. Body temperature was maintained at 37 ± 1°C by thermoregulated water via a circuit incorporated in the plastic bed. A respiratory sensor was then placed on the abdomen to continuously monitor respiration rate on a specialized device (ECG Trigger Unit HR V2.0, Rapid Biomedical).

MRI acquisitions were performed on a horizontal 7T Bruker Biospec MRI system (Bruker Biospin MRI GmbH) with a set of 400 mT/m gradients, controlled by a Bruker ParaVision 5.1 workstation. A Bruker birdcage volume coil (outer diameter = 112 mm and inner diameter = 72 mm) was used for signal transmission and a Bruker single-loop surface coil (25 mm diameter), positioned on the head of the animal to target the brain, for signal reception.

For the MRI protocol, 2D T2-weightedfat-saturated images (T2WI) on the rapid acquisition with relaxation-enhanced (RARE) method were obtained on axial slices. Acquisition parameters were as follows: echo time (TE) 60 ms, repetition time (TR) 5000 ms, RARE factor = 8, and average = 4. A total of 15.1 mm slices were acquired with field of view (FOV) of 3 cm × 1.5 cm and matrix size of 256 × 128, providing in-plane resolution of 117 × 117 microns, for 4 minutes' scan time.

#### 2.4.2. [^11^C]PIB PET/CT Imaging

[^11^C]PIB was labeled as previously described [21]. Radiochemical purity was >95%. After catheterization of a caudal vein, animals (*n*=4) were positioned prone in a micro-PET/CT apparatus (Inveon, Siemens) with the head centered in the field of view (FOV). Gaseous anesthesia was maintained via a cone mask, and breathing rate was monitored throughout the experiment. [^11^C]PIB with mean activity of 14.2 MBq (383 *μ*Ci) (range, 9.6–20.2 MBq) was injected intravenously as a bolus. Dynamic PET acquisition in list mode over 60 min was started immediately after radiotracer injection. CT scanning was performed to correct attenuation and scatter. All PET images were reconstructed by 3D ordinary poisson ordered subsets expectation-maximization (OP-OSEM3D) with 4 iterations and a zoom factor of 2. The reconstructed volume comprised 159 slices of 128 × 128 voxels, in a bounding box of 49.7 × 49.7 × 126 mm. Nominal in-plane resolution was ∼1.4 mm full-width-at-half-maximum in the FOV center.

#### 2.4.3. Image Analysis

**Figure 1:**
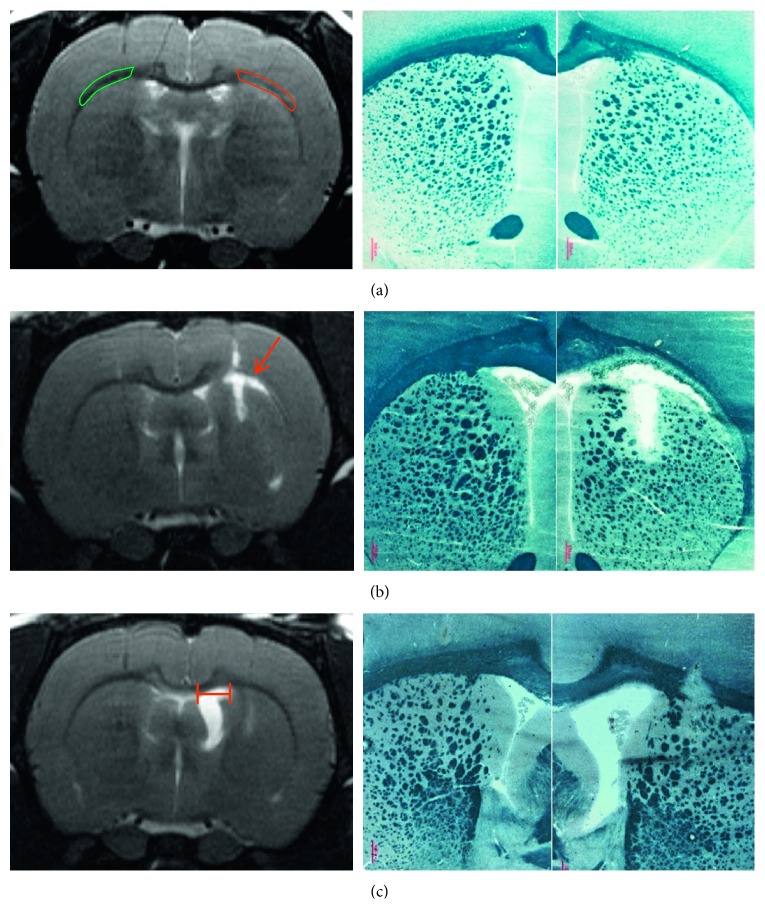
Comparison of MRI (T2WI, left column) and histology with Sudan black B (SBB, right column) at the injection sites. Postmortem histological staining matched *in vivo* MRI observations. Therefore, anatomical MRI was used to (a) manually delineate areas of demyelination showing corpus callosum loss of hypointense contrast (in red, with mirror region of interest in green), (b) identify necrosis areas with overt focal edematous hypersignal (arrow), and (c) measure the maximum width of the lateral ventricle along the mediolateral plane (red segment) as an index of ventricular dilation after LPC injection.

Using the MIPAV (Medical Image Processing, Analysis, and Visualization) application (https://mipav.cit.nih.gov/), MR images were visually inspected in search for areas in the corpus callosum exhibiting a normalization of the natively hyposignal (Figure 1(a)) and for any edematous hypersignal encompassing the corpus callosum and adjacent areas (Figure 1(b)). A region of interest (ROI) encompassing the abnormal area of the corpus callosum was manually drawn on MR slices and mirrored onto the contralateral corpus callosum. In addition, brain slices were screened to identify and measure the maximal width of the lateral ventricle along the mediolateral plane (Figure 1(c)). Each of these two measurements was performed by two operators (blind to other data). MR images were then imported in the Inveon Research Workpackage (IRW, Siemens) and registered onto PET/CT images. Summed tracer uptake (% injected dose per gram) in the ROIs was calculated from 20 to 40 minutes' acquisition.

### 2.5. *Ex vivo* Study

#### 2.5.1. [^18^F]AV-45 Autoradiography

[^18^F]AV-45 was labeled as previously described [22, 23]. Radiochemical purity was >95%. Under isoflurane anesthesia, animals (*n*=5) were intravenously injected with 12.6 MBq (340 *μ*Ci) (range, 8.6–18.9 MBq) [^18^F]AV-45 and euthanized 10 min after radiotracer injection. Brains were rapidly removed, snap-frozen at −20°C, coronally cryosectioned into 30 *μ*m slices, and mounted on glass slides. After air-drying at room temperature, slides were exposed to sensitive imaging plates (BAS-IP MS 2025, Fujiﬁlm) for 4 hours. The distribution of radioactivity was then digitized on a bioimaging analyzer (BAS-5000, Fujiﬁlm).

#### 2.5.2. Myelin Histological Staining

Following autoradiography, brain sections were postfixed with 4% formaldehyde in PBS, then briefly dehydrated in 70% ethanol. Slides were incubated in 0.1% Sudan Black B (SBB) solution (Sigma-Aldrich, ref. 199664) at room temperature for 10 min, washed in 70% ethanol for 10–30 s, then moved into distilled water for mounting in aqueous medium (Roti-Mount, Carl Roth). The slides with demyelinated lesions were observed and photographed under a microscope (Axioplan 2, Zeiss).

#### 2.5.3. Image Analysis

Autoradiograms were visualized on Multigauge software (Fujiﬁlm). ROIs were drawn manually on the targeted injection sites in the corpus callosum by a single operator, and lesion-to-contralateral uptake ratios were calculated. Corresponding ROIs were also drawn manually on histological images, and myelin content in ipsilateral and contralateral corpus callosum were semiquantitatively measured by an experienced observer blind to the autoradiography results, using Image-Pro Plus 6.0 software (Media Cybernetics) and expressed as optical density per unit area. The lesion-to-contralateral ratios were then calculated for optical density per unit area. For each animal, quantification was performed on 4 brain sections encompassing the whole volume showing a decreased binding, hence resulting in 20 measurements.

### 2.6. Statistical Analysis

Data were analyzed on SPSS 19.0 software. Group comparisons were performed using Kruskal–Wallis tests and Mann–Whitney test after binarization of side-effect detection. Slice-by-slice correlation between autoradiography and histology measurements, as well as correlation between operators, used Spearman's tests. The significance threshold was set at *p* < 0.05.

## 3. Results

### 3.1. MRI-Based Optimization of LPC Injections

Optimization of the injection protocol was driven by the need to obtain a large area of demyelination in the corpus callosum, so as to be clearly detected *in vivo* on PET. However, necrosis and adjacent ventricular dilation are pitfalls commonly reported after LPC injection [12, 18–20]. In line with these reports, our first attempts to establish a pure model of demyelination highlighted the need to keep a low LPC concentration (1%) and low injection speed (0.1 *μ*L/min) (data not shown). Because visual postmortem examination of brain tissue, seen upon cutting brains on a cryostat, may be biased by extraction and processing, *in vivo* anatomical T2-weighted MRI was used to evaluate different injection protocols. Although T2 contrast might be influenced by several concurrent processes, pilot histological comparisons showed a fair agreement between (i) the loss of the natively hypointense contrast of corpus callosum and successful demyelination (Figure 1(a)) and (ii) strong edematous hypersignals and necrosis (Figure 1(b)). Therefore, in an effort to provide immediately available criteria for enrolling animals into a subsequent PET protocol, the following simple MRI metrics were used for evaluating the optimization process: (i) manual delineation of signal abnormality on corpus callosum as a surrogate for demyelination (Figure 1(a)) and (ii) manual measurement of the maximal width of the lateral ventricle as a surrogate for abnormal dilation (Figure 1(c)). Two operators independently performed these two measurements with overall good reproducibility (both correlations were significant at the *p* < 0.01 level). Importantly, deviations were below the resolution of PET imaging. Raw data are provided as supplementary dataset S2, and mean of the two measurements is reported thereafter.

**Figure 2:**
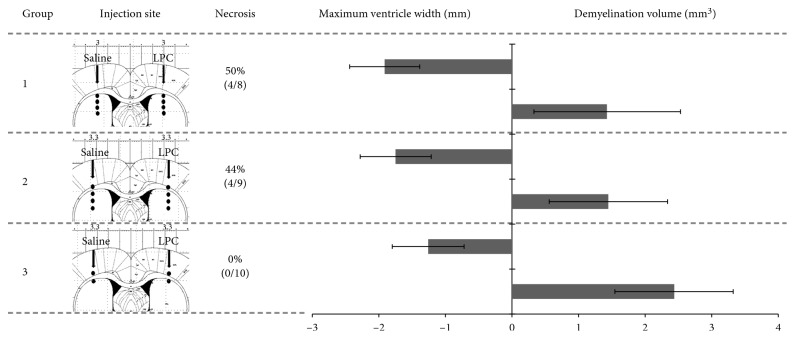
Optimization of the LPC injection protocol. LPC concentration (1% in saline) and infusion rate (0.1 *μ*l/min) were kept constant between the three groups. The injection sites are shown in the corresponding Paxinos coronal diagram, with the following coordinates: group 1, AP −0.3 mm, ML ±3.0 mm, DV −3.5/−4.0/−4.5/−5.0 mm; group 2, AP −0.3 mm, ML ±3.3 mm, DV −3.0/−3.7/−4.3/−5.0 mm; group 3, AP −0.3 mm, ML ±3.3 mm, DV −2.8/−3.5 mm. Each site was infused with 2.5 *μ*l of LPC, from depth to superﬁcial. The rate of animals exhibiting focal edematous hypersignals on MRI (as in Figure 1(b)) is given as a percentage (and number of animals out of total the group number). The graph shows the maximum ventricle width (measured along the mediolateral plane as in Figure 1(c), and arbitrarily expressed as a negative value, in mm) and the total volume of corpus callosum exhibiting a normalization of the natively hypointense contrast (measured as in Figure 1(a), in mm^3^).

Figure 2 summarizes the results of this optimization process in three experimental groups. In the first group, we adapted previously reported injection conditions (group 1: 4 injection sites in striatum and corpus callosum; total volume 10 *μ*l). This led to detectable edematous hypersignals in half of the animals and a mean ventricle width of 1.9 ± 0.5 mm. In group 2, increasing the distance between the 4 injection points and the lateral distance from the bregma only slightly decreased the rate of edema (4 over 9 animals) and reduced ventricle width (1.7 ± 0.5 mm). As edematous foci were mainly observed in the striatum, we simplified the injection protocol and kept only two injection sites, at the lower and upper levels of the corpus callosum (group 3; total volume 5 *μ*l); with this protocol, no animals showed focal edema, and ventricle width was further reduced (1.2 ± 0.5 mm). The mean volume of the abnormally normalized signal in the corpus callosum increased in parallel with the reduction of LPC side effects, reaching 2.4 ± 0.9 mm^3^, which suggested increased demyelination. However, these measurements were not significantly different between groups (*p* > 0.05), highlighting the residual variability of the model and prompting us to examine how PET signals were affected. Would the following exclusion criteria have been applied: (i) focal edematous hypersignal or (ii) maximal lateral ventricle width >1.4 mm (corresponding to the in-plane PET resolution)—only 5 animals over 27 would have been considered devoid of side effects and selected (1 in group 1 and 4 in group 3). Of note, the volume of the abnormally normalized signal in the corpus callosum, or “apparent” demyelination, was significantly higher in these 5 rats than in the 22 others with at least one side effect (*p*=0.03).

### 3.2. *In vivo* [^11^C]PIB PET

**Figure 3:**
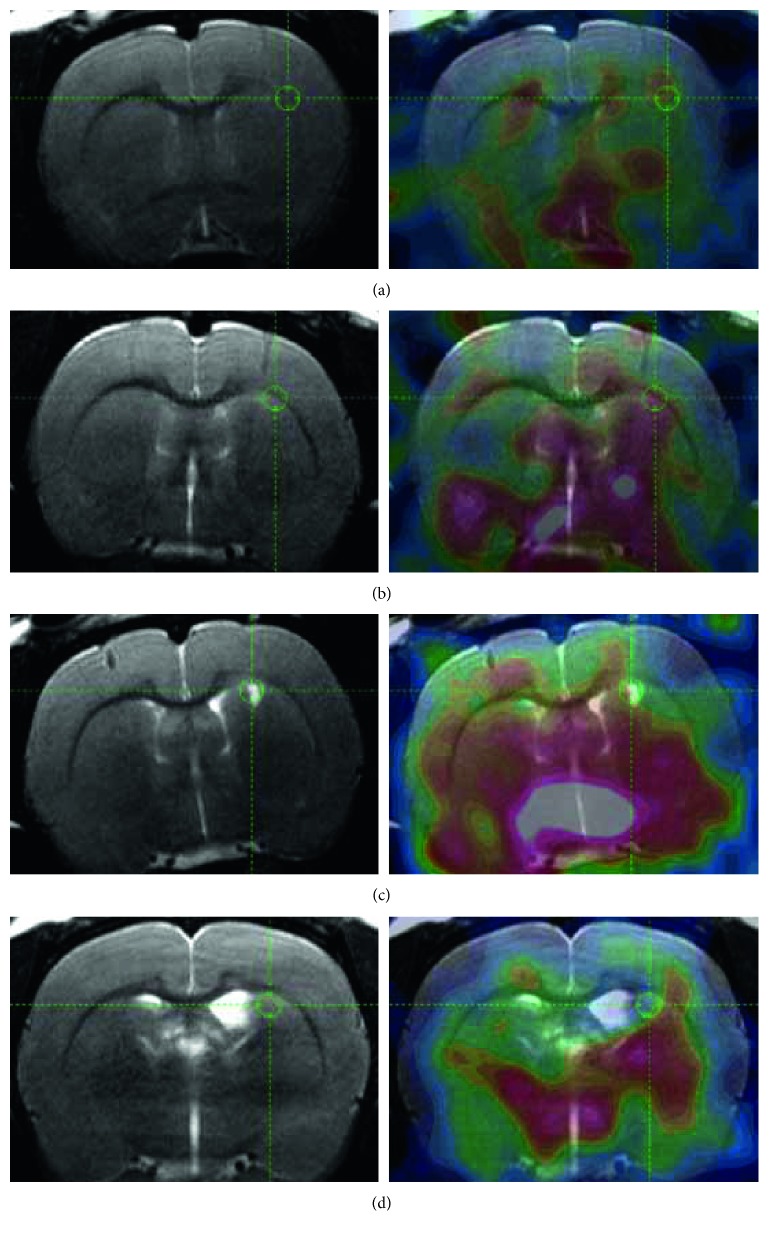
*In vivo* PET imaging with [^11^C]PIB. T2WI MRI is shown in the left column and overlaid with a 20 min summed PET image of [^11^C]PIB in the right column. Rats A and B (a, b) exhibited a large demyelination area without necrosis or ventricle dilation. Rats C and D (c, d) presented a smaller demyelination area and necrosis (c) or ventricle dilation (d).

Among a new batch of animals, injected in the conditions as group 3, four additional rats were selected, to reflect the variety of lesions and pitfalls following LPC injection. These animals underwent [^11^C]PIB PET imaging between 8 and 15 days after stereotaxic injection. Apparent demyelination volume on MRI and [^11^C]PIB uptake within this volume and in a mirror volume in the contralateral corpus callosum are reported in Table 1. In rats A and B, [^11^C]PIB uptake was not decreased (ratio ≥ 1) despite a large demyelination area without edematous lesion or ventricle dilation (Figures 3(a) and 3(b)). Rats C and D presented a smaller demyelination area and one side effect each: focal edema in rat C (Figure 3(c)) and ventricle dilation (max width > 1.4 mm) in rat D (Figure 3(d)). MRI-driven quantification showed slightly decreased [^11^C]PIB uptake in rat C (ratio 0.88) but not rat D (ratio 1.00). Importantly, PET images highlighted decreased PIB uptake at the necrosis and ventricle dilation sites. These results strongly suggested that *in vivo* [^11^C]PIB PET imaging could not reliably detect demyelination in the LPC-induced rodent model. Moreover, side effects of LPC injection may lead to false-positive detection of demyelination when concurrent MRI is not available. These qualitative but clear-cut results were considered as an endpoint for the PET study.

### 3.3. *Ex vivo* [^18^F]AV-45 Autoradiography

**Figure 4:**
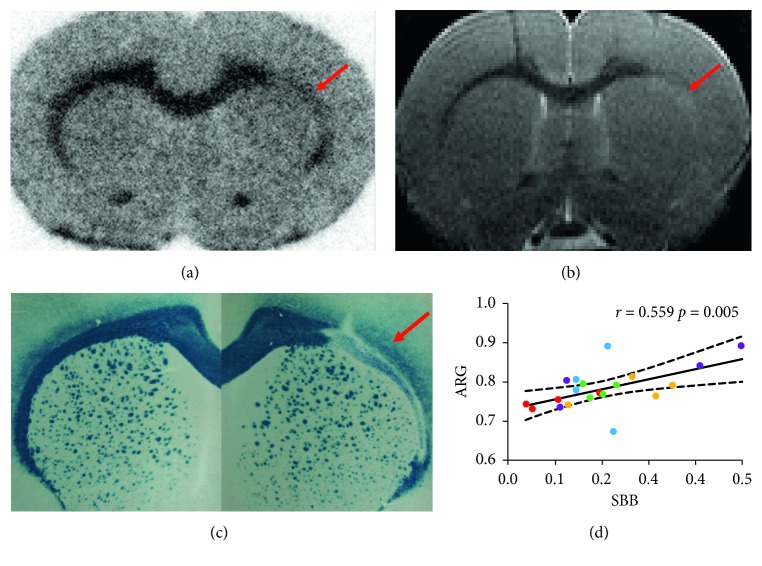
*Ex vivo* autoradiography with [^18^F]AV-45. Reduced [^18^F]AV-45 uptake in the LPC-injected site was visually identified ((a), arrow), and matched T2WI MRI (b), as well as Sudan Black B staining, which confirmed demyelination (c). Four sections per animal (each rat is represented with a different color), encompassing the whole area showing a decreased binding, were analyzed, hence resulting in 20 measurements (d). There was a significant correlation (*r* = 0.559, *p*=0.005) between the ratio of ipsi-to-contralateral [^18^F]AV-45 binding on autoradiography (ARG) and the corresponding ratio of ipsi-to-contralateral optical density on Sudan Black B (SBB) staining (plain line, linear fit; dashed lines, 95% confidence interval).

Because *in vivo* detection of LPC-induced demyelination may be inaccurate due to lack of spatial resolution and consequently decreased sensitivity, 5 additional animals, injected in the same conditions as group 3, underwent *ex vivo*high-resolution autoradiography. Obtaining *ex vivo* images with a measurable signal-to-noise ratio required changing from the carbon-11 PIB tracer to a fluorine-18 radiotracer, such as [^18^F]AV-45. At this 100 *μ*m spatial resolution, pitfalls of the animal model were easily identified as complete lack of signal in the 2D images (Supplementary Figure S3) and could not be confounded with loss of binding in the demyelinated corpus callosum. Binding in the ipsilateral corpus callosum was clearly decreased in all animals (Figure 4(a)), confirming the MRI observations (Figure 4(b)). The ipsi-to-contralateral [^18^F]AV-45 uptake ratio, averaged from 4 brain sections per animal, was similar in all five animals (0.78 ± 0.02). Furthermore, subsequent myelin histology on the same sections correlated visually (Figure 4(c)) and quantitatively (Figure 4(d), *r* = 0.559, *p* = 0.005) with the corresponding [^18^F]AV-45 signals.

## 4. Discussion

Unilateral LPC-induced demyelination has gained increased popularity as a first-line animal model for preclinical evaluation of imaging biomarkers. Compared to other rodent models of demyelination, it has the advantages of (i) producing larger demyelination lesions than EAE models [24] and (ii) not requiring another group of control animals, as for transgenic shivered mice [25] or cuprizone-induced demyelination [26]. The goal of the present study was to set up a workflow for the evaluation of new myelin radiotracers using this LPC model in rats.

As a first step, we observed, as previously reported, that demyelination of the corpus callosum can be accompanied by necrosis and/or ipsilateral ventricle dilation [19, 20]. Necrosis might be due to locally excessive LPC concentration, and ventricle dilation is thought to be mediated by inflammation. Here, we used anatomical MRI to monitor the incidence of these side effects *in vivo* (Figure 1). By targeting white matter in the corpus callosum only (without striatum), and by increasing the mediolateral distance of the injection sites, we were able to reduce the proportion of animals without any side effects. Further refinements of the injection procedure might include the use of (i) glass-capillary microneedles to minimize tissue damage and nonspecific inflammatory responses [27] and (ii) T2 mapping instead of T2-weighted imaging, so as to allow an operator-independent,threshold-based, estimation of ventricle volume, and corpus callosum apparent demyelination [28].

Although previous studies reported testing several injection conditions [17, 19], the impact on imaging was never assessed. Therefore, in the second step, a limited number of PET imaging sessions with the reference radiotracer [^11^C]PIB were conducted in additional animals representing the range of pathological conditions observed after LPC injection. The results unambiguously showed that ipsilateral tracer uptake in areas of demyelination was not decreased after LPC injection (rats A and B), although their volume exceeded the resolution of the small-animal PET scanner. Even more concerning was the observation of apparently decreased uptake in the necrosis site or enlarged ventricle (rats C and D). Therefore, in the absence of individual MRI, PET-driven analysis might incorrectly suggest demyelination (false-positive detection). These results highlight the low sensitivity of [^11^C]PIB for detecting demyelination in small-animal models. Several factors may be put forward, including the mm-range resolution of small-animal PET scanners, combined with the low volume of highly myelinated axons in rodents, but also the relatively high nonspecific binding of [^11^C]PIB. For ethical reasons, we considered these qualitative but clear-cut results as an endpoint for our PET study.

In the third step, we used *ex vivo* autoradiography instead of *in vivo* imaging. Five additional animals were injected with the fluorine-18 radiotracer [^18^F]AV-45, because the short half-life of carbon-11 prevented accumulating enough signal. It should be noted that *in vitro* autoradiography is of little value for assessing radiotracer binding to myelin, because white matter to gray matter contrast entirely depends on washing conditions (data not shown) and might not reflect *in vivo* uptake. *Ex vivo* autoradiography appeared to be a viable strategy for assessing radiotracer performance in the LPC model for several reasons. First, side effects were easily identified and distinguished from the surrounding tissue on brain sections. Second, the signal drop in the injected corpus callosum reached 20%, which was highly reproducible (coefficient of variation < 3% between the 5 rats) and correlated with histology measurements. Though quantification was restricted to discrete 2D measurements on four brain sections per animal in this proof-of-concept experiment, 3D-reconstruction methods dedicated to autoradiography may be used in future studies to assess signal drop in a continuous volume similar to *in vivo* imaging [29]. Overall, these promising results are in line with recent reports of repurposed fluorine-18 labeled amyloid radiotracers in MS patients (florbetapir or [^18^F]AV-45 [30] and florbetaben or [^18^F]AV-1 [31]).

## 5. Conclusion

This study aimed to draw attention to common pitfalls associated with LPC injections in the central nervous system and their impact on nuclear imaging of myelin. While this animal model is attractive for evaluating imaging biomarkers of demyelination and remyelination, *in vivo* PET imaging in small animals may be sensitive to side effects of LPC injections rather than real demyelination. We conclude that appropriate use of this rodent model requires MRI to correctly identify animals with pure demyelination and *ex vivo* autoradiography to track spatial myelin changes with enough sensitivity. Alternatively, longitudinal studies with *in vivo* PET imaging could possibly be performed after LPC injection in larger animals, such as rabbits [32], swine [33], or primates [34].

## Figures and Tables

**Table 1:** Quantification of [^11^C]PIB uptake in ipsilateral (LPC) and contralateral (SAL) regions of interest (ROI) manually drawn onto T2WI (as shown in Figure 1(a)). The LPC-to-SAL ratio is expected to be < 1 in case of demyelination.

Rat	MRI observations	Vol. (mm^3^) of ROI on T2WI	[^11^C]PIB uptake (%ID/g)		
			LPC	SAL	Ratio
A	Large demyelination	5.20	0.16	0.15	1.03
B	Large demyelination	3.70	0.53	0.47	1.12
C	Small demyelination with necrosis	2.20	0.25	0.29	0.88
D	Small demyelination with ventricle dilation	1.90	0.23	0.23	1.00

## Data Availability

All the data used to support the findings of this study are available from the corresponding author upon request.

## Supplementary Materials

Supplementary Materials Document S1: completed “The ARRIVE Guidelines Checklist” for reporting animal data in this manuscript (downloaded from https://www.nc3rs.org.uk/arrive-guidelines). Dataset S2: individual measures and observations performed in rats from groups 1–3, by two independent operators. Figure S3: *ex vivo*[18F]AV-45 autoradiography sections showing the visual identification (red arrows) of cavitation resulting from necrotic tissue (A) and ventricle dilation (B).
